# The Mediating Role of Psychological Empowerment on the Relationship Between Digital Transformation, Innovative Work Behavior, and Organizational Financial Performance

**DOI:** 10.3390/bs15010005

**Published:** 2024-12-26

**Authors:** Saqib Muneer, Ajay Singh, Mazhar Hussain Choudhary, Awwad Saad Alshammari

**Affiliations:** 1 Department of Economics and Finance, College of Business Administration, University of Ha’il, Ha’il 55476, Saudi Arabia; 2 Department of Management and Information Systems, College of Business Administration, University of Ha’il, Ha’il 55476, Saudi Arabia

**Keywords:** digital transformation, organizational financial performance, psychological empowerment, IWB

## Abstract

With the use of digital technologies in today’s innovation era, the financial sector has transformed how they facilitate customer business and generate a high level of revenue. This study aims to explore the relationship between innovative work behavior (IWB), digital transformation (DT), and organizational financial performance (OFP) to analyze the mediating role of workers’ psychological empowerment (PE) between independent and dependent variables. Further, we examined the moderating role of smart technologies (ST) between PE and OFP. This study collected data from Saudi banking sector employees using a well-structured questionnaire adopted from previous literature. Next, Smart-PLS was used to analyze the data using the structural equation modeling-partial least squares (SEM-PLS) approach. The results reveal that IW positively relates to OFP, with PE mediating this relationship. Furthermore, DT positively affects OFP. ST acts as a positive moderator that enhances workers’ PE and OFP. Meanwhile, PE, as a mediator, positively relates DT and IWB to OFP. Overall, this study makes valuable theoretical, empirical, and practical contributions, which can benefit bank management, policymakers, and future academic research.

## 1. Introduction

Digital transformation (DT) has significantly reduced international boundaries by fostering interconnectivity between countries. These global interactions have introduced new momentum to the market (Yasin et al., 2022). For instance, DT has not only altered the way of life through which individuals communicate with each other and their surroundings, but also digitally transformed production processes. DT advancements have affected all aspects of business (Cheng et al., 2020).In the context of business growth, DT has strengthened competition globally. To compete well, enterprises increasingly utilize technology in production and marketing. In addition to influencing the business landscape, DT has changed people’s living standards. It offers significant prospects for creating a well-organized market structure. Crucially, a vital contribution of DT is seamlessly connecting the service provider with the service user with the help of innovative technology (ST) (Cheng et al., 2020).

Further, DT has also played a significant role in the service sector, such as the banking sector. For instance, it has improved banking services by introducing online banking, digital wallets, and mobile apps. These banking sector advancements have provided customers with a convenient and optimal experience (Pramanik et al., 2019). Furthermore, utilizing data analytics and artificial intelligence, DT helps personalize banking services and gives customers a choice based on their preferences. DT has also reduced paperwork and manual tasks and enhanced working efficiency (Ghobaei-Arani et al., 2020).

In contrast, DT has important effects on organizational performance, while employees’ innovative work behavior (IWB) is also crucial for attaining competitive benefits from DT in the long run. IWB comprises generating, intensifying, and incorporating ideas within the organization. It also contributes to improving overall business value. IWB enables organizations to remain competitive by adapting to market changes, driving innovation, and responding more effectively to customer needs. Furthermore, organizations that cultivate a culture of innovation through employee behavior improve their operational processes and experience more significant long-term growth and sustainability. Therefore, IWB has become imperative across businesses and industries to enhance firm performance (Liedtka, 2018).

Many studies (Ruhl, 1995; Giatsidis et al., 2019; Garay-Rondero et al., 2020; Kamselem et al., 2020; Gassmann et al., 2020; Chapple et al., 2020; Rumjaun & Narod, 2020; Song et al., 2022; Ancillai et al., 2023; Sarkar et al., 2024) have explored the DT, IWB, OP, and technologies. However, some research gaps remain. For example, these studies did not delve into essential features, such as market share and earnings growth, or use different dependent variables. These studies have limitations regarding variables, data, and methodology. A study by Giatsidis et al. (2019) relates DT with information technologies in banking industries; this study used self-efficacy, eases of use, etc., as variables and suggested further study on the financial sector. Another study by Garay-Rondero et al. (2020) explained the dimensions of the digital supply chain through a qualitative meta-analysis and developed an industrial-level model. However, this study did not provide any practical implementation of these dimensions, leaving a gap in the understanding of how these theoretical concepts can be applied in the real-world financial sector. Furthermore, one study Bellandi et al. (2019) argues that the DT dimension lacks practical implications, especially in the financial sector of developing countries such as Saudi Arabia. Crucially, these studies lack consistent conclusions, implying the need for empirical analysis using advanced analytical tools such as Smart-PLS version 4.

Therefore, the current study aims to determine DT and IWB’s collective impact on OFP in the Saudi banking sector. Further, the mediating role of psychological empowerment between IWB and DT with OFP should be investigated. Moreover, this study examines ST’s role in the moderation between PE and OFP. Overall, this study offers significant insights into the key factors that contribute to the success of firms as they navigate the challenges of the digital era. Understanding the impact of IWB and DT on financial performance provides valuable information for making business decisions and practical advice on how innovation and digitalization can be effectively used to meet financial goals. Furthermore, this study provides a detailed understanding of the mechanisms underlying these associations through a mediated moderating model where workers’ psychological empowerment (PE) is a mediator, and ST is a moderator. This comprehensive approach makes theoretical contributions and provides practical insights for legislators, administrators, and practitioners who want to succeed in today’s ever-changing business environment. Furthermore, this study offers significant insights into the critical pathways for achieving organizational success amid the disruptive effects of the digital ecosystem. As digital transformation reshapes industries, organizations must navigate challenges while capitalizing on opportunities for growth and innovation. This study identifies key factors, such as psychological empowerment, innovative work behavior, and financial performance that contribute to resilience and competitiveness in a digital landscape. This research highlights the importance of aligning digital initiatives with workforce empowerment strategies by demonstrating how digital tools enhance employee autonomy, flexibility, and decision-making.

Furthermore, this study bridges a gap in the existing literature by examining the mediating role of psychological empowerment, an area previously underexplored in digital transformation. Previous studies have focused on technological adoption and performance outcomes; however, this research offers a more holistic view by incorporating the human aspect of digital transformation. This comprehensive approach provides valuable insights for policymakers seeking to create supportive digital innovation and organizational growth environments. Additionally, it lays the groundwork for future research, encouraging scholars to explore the longitudinal effects, cross-industry comparisons, and evolving role of leadership in fostering digital transformation-driven success (Kamselem et al., 2020).

## 2. Literature Review and Theoretical Framework

This article discusses related theories, such as business model innovation and social learning theory (Kamselem et al., 2020). A business model outlines a business’s core operations and value-creation mechanisms. This theory states that changing one’s mind or reforming these components will improve current processes and principles (Ruhl, 1995). This may need developing technologies to enhance novel policies. It encompasses various functions and offers novel business strategies, administrative structures, trading practices, and operational policies. Successful business model innovation depends on leveraging technologies in novel ways. It involves strategically deciding whether to overhaul the entire model or reinvent specific components. This approach can be a powerful tool for navigating business uncertainties when implemented appropriately and at the right juncture (Gassmann et al., 2020).

From the perspective of DT, BMI acts as an antecedent that brings the solution that organizations follow and exploit digital instruments (Ancillai et al., 2023). OFP works as an initial antecedent as financial stability may work as a promoter for organizations to invent their business model to continue or develop turnover rates. ST also works as an essential antecedent by providing instruments that allow business model innovation. PE is vital as a cognitive antecedent when workers feel free and adopt new methods to explore new ideas. BMI theory redesigns important components like profit models, in response to digital modifications. Excellent attention to workers’ PE inspires the use of new technologies or innovative applications. ST, like artificial intelligence and independence in innovative technologies, encourages workers to improve processes, enhance consumer collaboration, and increase digital profit rates. Innovative technologies also moderate this process by allowing data to provide insights and productivity (Mubarak & Petraite, 2020).

Social Learning theory, developed by Albert Bandura, describes the role of observation and exhibiting in learning. It represents the behavior of the people they receive from others and replicates their actions (Rumjaun & Narod, 2020). Social learning theory delves into how intellectual processes affect learning. The reciprocal determinism concept by Bandura shows the connection between behavioral and social elements in defining acts. Therefore, social learning theory gives significance to the social context of mortal behavior and progress. Social learning theory describes the learning process by analyzing others, including leaders. In DT, workers emulate behaviors such as using innovative technologies. Leaders and trained workers bring attention by shaping new instruments, which PE reinforces. Innovative technologies support learning through analytical profits and higher collaboration in digital transformation. This act improves the capability of OFP, accelerating the transformation progress (Sarkar et al., 2024).

Moreover, Bandura’s social cognitive theory is another essential aspect of this study’s context, and it was proposed in 1960. This theory explains that employees obtain more information through task information and the external impact of social media explanations or associations. Social learning theory informs about learning dynamics, social dynamics, conservational issues, and personal influences that effectively inspire individuals to accomplish actions within an organization. In addition, this theory postulates that individuals must adequately manage information through processes such as dialogue, conversion, and recombination of concepts (Chapple et al., 2020).

Furthermore, this study includes social cognitive theory and highlights the behavior of self-productivity and analytical learning design. PE is a mediator that increases workers’ confidence level in absorbing DT. ST works as a moderator, shaping an atmosphere where analyzing the progress of others with the use of this instrument and encourages identical behaviors. Some outcomes involve enhanced DT or OFP. Trained workers or the best use of innovative technologies promote innovative behavior and culture. These modifications improve progress, decrease expenditure, or bring about continuous development and confirm long-term progress (Song et al., 2022).

### 2.1. Digital Transformation

Organizations use DT to implement and execute digital technologies. DT creates and adjusts recent products and procedures to translate business processes into digital formats. DT helps businesses improve and redesign their business models for implementation and inflexibility in an energetic marketplace. Firms use the DT process to reshape significant formations by implementing digital technology. DT also allows consumers and suppliers to negotiate costs and revenue using information and self-confidence (Bellandi et al., 2019).

Some antecedents are included in DT, such as technological advancement, organizational ability, and innovation capabilities (Mubarak & Petraite, 2020) Technological advancements, including artificial intelligence and unique ideas, provide equipment that allows organizations to reconfigure their work and enhance significance distribution. For example, organizations may operate effectively, while artificial intelligence determines the results to improve decision-making ability. Moreover, innovation capabilities are the ability to provide new suggestions that are important in leading to business fluctuations. For instance, Tesla’s innovation of incorporating smart technologies into electric vehicles has recreated consumer expectations and increased financial development (Turner et al., 2020).

The consequences of DT include improved DFP and higher consumer satisfaction. DT can increase profits and development. For example, the seamless integration of Amazon’s tools into consumer facilities has contributed to its marketplace and profit progress. Some outcomes of DT include an increase in working efficiencies—experienced customers with personalized services and some advanced technologies (Maddikunta et al., 2022).

Therefore, DT is used in the banking sector to help banks modernize operations and enhance consumer experience. Banks adopt DT technologies such as mobile banking systems, promotion of financial presence, and advancing consumers’ wants in the rapidly progressing digital countryside of Saudi Arabia (Giatsidis et al., 2019).

### 2.2. Innovative Work Behavior

IWB tried to include the practical recreation of new ideas, techniques, and explanations in work situations. Innovative work performance fosters a culture of experimentation and creativity, where all employees feel free and have the liberty to think outside and challenge conformist methodologies (Kraft, 2018). In reality, in controlled industries like banking, innovation, compliance improvement, and customer service are more important rather than unrestricted employee creativity. The bureaucratic structures in the banks hinder workers’ ability to see outside the box. The rigid organizational structures suppress innovative abilities and behavior (Ma & Zhao, 2016). Therefore, IWB is vital for maintaining business significance in the banking sector in the age of DT. The banking sector is discovering new technologies to improve consumer experience and moderate threats. By adopting an IWB process, banks can incorporate changing marketplace dynamics and continue to compete in the financial market (Fenwick & Vermeulen, 2020).

### 2.3. Psychological Empowerment

PE is an essential practice in business administration. PE provides employees with confidence and resources to address challenges and improve output and invention (Azlan & Wahab, 2020). An innovative process empowers the workforce, which increases innovative practices by allowing decision-making or knowledge. This approach provides employees with all the resources needed to improve work productivity or increase individuality in their work involvement (Obiekwe et al., 2019). PE is vital for enhancing productivity and innovation in the banking sector. PE allows the banking system to make professional and well-informed decisions, offer superior accountability, and adapt to the dynamic background of financial facilities, which are essential for improving customers’ sustainable progress and knowledge (Almulhim, 2020a).

### 2.4. Smart Technologies

STs involve critical processes that match the innovative structure of DT. The integration of STs leads to innovations in management practices (Ma & Zhao, 2016). Some characteristics, such as programmability or sustainability in somatic policies, allow organizations to keep pace with marketplace fluctuations and satisfy customers. Technologies control digital innovation to increase user capabilities in various industries (Urbinati et al., 2020). Further, ST is essential for restoring traditional banking processes and consumer interactions in the banking sector. They allow banks to update their procedures and improve their financial goods.

### 2.5. Organizational Financial Performance

Organizations tackle different internal and external challenges throughout the world. They must simultaneously improve compliance, openness, and invention, both externally and internally. Organizations need rapid development for enduring learning, transitioning from a creative-centric method to a more efficient one. Teamwork with stakeholders to improve innovation and actions at the executive level is vital to this conversion (Corsino et al., 2019).

OFP shows an organization’s overall progress in fulfilling its tactical purposes. Financial performance is classically measured by significant indicators such as productivity, liquidity, capital worth, and asset capability (Almulhim, 2020b). Productivity measures with return on assets (ROA) and return on equity (ROE), which convey the ability of the bank to produce income from its resources or equity capital, respectively. In short, driven financial performance in the banking sector is vital for building investor confidence, issuing securities, and ensuring secondary sustainable development and invention.

### 2.6. Hypotheses Development

#### 2.6.1. Digital Transformation and Organizational Financial Performance

In recent years, DT has transformed business processes (Butt, 2020), where technological improvement has simplified and required management to improve production levels and enhance connections with numerous investors to distinguish the organization in terms of service provision. Many studies underline the transformative influence of technological innovation, considering a strong driver of creativity toward superior marketplace effectiveness and exceptional income generation. Approval innovation and integrating technological conversion into invention- and package-providing monarchies have become essential for organizational existence and well-being (Del Castillo, 2018).

Moreover, practical DT efforts can enhance the financial outcomes of firms in diverse sectors. Organizations using digital technology effectively achieve incredible sales growth and profitability compared to their less digitally mature competitors (Giatsidis et al., 2019). Furthermore, one study by Obiekwe et al. (2019) provides evidence of a direct correlation between investments in digital technology and firm productivity. These results emphasize the significant impact of digitization on enhancing an organization’s competitiveness and financial performance.

Furthermore, the literature indicates that the influence of DT on financial performance relates to aspects such as organizational agility, innovation capabilities, and customer experience Obiekwe et al. (2019). In particular, being adaptable and flexible is essential for effectively managing digital disruptions. Similarly, another study Corsino et. al. (2019) showed that innovation skills are crucial for connecting DT and financial success. This finding suggests that businesses with high innovation ability are better positioned to take advantage of digital opportunities and achieve long-term growth. In general, the literature highlights the complex connection between DT and OFP. This emphasizes the importance of comprehensive approaches that consider technological investments and organizational skills. Therefore, we propose the following hypothesis:

**Hypothesis 1.**  *DT has a positive relationship with OFP*.

#### 2.6.2. Digital Transformation and Psychological Empowerment

DT eases the rapid conversion of information (Bin Saeed et al., 2019)Using DT, employees can educate themselves well about manufacturing tendencies, business information, and best performance, enabling them to make well-intentioned contributions and express their efforts (Hertel-Fernandez, 2018). Instruments for DT affect employees’ PE. Moreover, digital collaboration instruments encourage statements and limpidity, which are prominent features of a compassionate work atmosphere (Yasin et al., 2022).

However, it can result in issues such as digital excess or continuous association, which are hypothetically important for increased pressure and stress. Actual applications of DT involve organizations reporting these concerns through bold, acceptable training, educating a culture of psychological security, or promoting strong digital practices. Eventually, the effect of DT on employee empowerment becomes complicated, necessitating a stable method to exploit its benefits and address possible weaknesses (Saarikko et al., 2020).

Some researchers have investigated the impact of digital technology on how employees perceive empowerment and participation in digitalized work environments Corsino et al. (2019). For example, one studies Corsino et al. (2019) showed that digital technologies can increase employees’ feelings of empowerment by granting them access to information, resources, and decision-making tools.

Moreover, studies indicate that PE is essential for facilitating the connection between DT and organizational results, such as performance and innovation (Corsino et al., 2019; Urbinati et al., 2020). This emphasizes the significance of psychological variables in effectively translating digital investments into organizational success. One study Ahsan (2024) discovered that PE mediates the relationship between transformational leadership and employee creativity. Generally, the literature indicates a mutually beneficial relationship between DT and PE. This means that digital technologies help improve employees’ feelings of empowerment, increasing organizational effectiveness, and innovation. DT and PE involve information accessibility, decision—making instrument availability, and digital stages of collaboration (Lim et al., 2020a). Empowered by workers with digital instruments and capital, digital transformation creates an atmosphere where workers feel empowered and more informed about all decisions (Zhen & Ding, 2024). —for example, instruments such as collaboration and Microsoft Teams allow workers to complete their tasks. Organizations with working environments foster openness and are more likely to strengthen these uses, as workers feel free to use digital capital more efficiently (Trenerry et al., 2021).

Empowered workers can find solutions to problems, contribute to digital innovation, and align their struggles with an organization’s planned aims (Gierlich-Joas et al., 2020). For instance, research has explained that PE mediates the link between transformational leadership and employee creativity, saying that digital instruments can create new ideas for effective employees (Khan et al., 2020).

Accordingly, we propose the following hypothesis:

**Hypothesis 2.**  *DT has a positive relationship with PE*.

#### 2.6.3. Psychological Empowerment and Organizational Financial Performance

Psychological empowerment describes the ability of inspiration and control that workers experience in their role and has positively affected organizational financial performance (Afram et al., 2022).When workers feel free, they identify their work as significant, trust that they can make progress, and learn the effect of their struggles on the organization (Garay-Rondero et al., 2020). These elements give peak levels of job satisfaction and obligation to the organization. Working and empowered workers like to go up and down in their work tasks, and problem-solving ideas can inspire enhanced development or recital consequences (Jaman et al., 2022).

An organization that promotes psychological empowerment likes to experience more innovation and productivity, as workers make decisions for their tasks, which helps the organization perform well (Safari et al., 2020). Moreover, empowered workers are more inspired to participate in the organization’s progress, teamwork, and success. Revenue rates have also determined a positive relationship between psychological empowerment and financial performance (Kraft, 2018). Authorized workers feel empowered by their work and devoted to the organization, including low absence and profit rates. This decreases employment and supports retaining an established workforce, donating more to financial development (Rodríguez-Sánchez et al., 2020).

Additionally, psychological empowerment has a positive relationship with organizational financial performance and shows that when workers feel free, improved commitment and retention support bring about organizational development. Organizations can create a valuable working atmosphere for businesses and each person by highlighting workers’ authorization (Füzi et al., 2022).

Moreover, some antecedents like proficiency, significant work, independence, and observed effects have been described (Kadam et al., 2020). When workers are empowered to make decisions, they feel free and have logic of authorization. Organizations promote a culture of belief, give chances for skill progress, and identify the impact on workers (Akdere & Egan, 2020). In this case, if an organization inspires workers to make decisions related to projects and creates an atmosphere that encourages authorization, independence in decisions strengthens this, as workers identify their starring role as essential to the success of an organization. The consequences of PE are marked by improved OFP, innovative work behavior, or employee retention (Sjahruddin et al., 2024).

Furthermore, working employees determine their job satisfaction, which leads to enhanced productivity. For instance, employees show solutions to problems and participate in good organizational consequences. Moreover, psychological empowerment positively relates to financial performance by decreasing absence and profit rates (Afram et al., 2022). Workers who feel empowered are less likely to leave and are experienced work teams. Organizations such as Zappos, which identify workers’ power and independence, have shown essential enhancements in consumer satisfaction and profit development (Anisdahl & Lerberg, 2020).

**Hypothesis 3.**  *PE has a positive relationship with OFP*.

#### 2.6.4. Psychological Empowerment as a Mediator Between Digital Transformation and Organizational Financial Performance

As a mediator, PE determines the link between DT and OFP (Liu et al., 2019). DT, which incorporates innovative technologies and procedures, can improve organizational productivity, inventions, and flexibility (Gimpel et al., 2018). However, industrial dynamics describes how financial improvements are used to observe how employees implement these changes. PE is a mediator that promotes employee involvement with or influences digital instruments. When employees feel allowed to work in a digital place, experience self-sufficiency, identify the significance of their task, or require the adjustment of instruments, they are more likely to incorporate the changes carried out by DT (Ma & Zhao, 2016).

Therefore, employees become more psychologically empowered and well-suited to implementing the full possibilities of digital technologies, and they are funded to improve the quickness and productivity of an organization. Finally, employee empowerment is a serious issue in mediating the effect of DT on the OFP; it stimulates the workforce to use digital instruments to realize productivity gains and adequate returns. Business intelligence instruments allow employees to view performance metrics and marketplace tendencies. This information will enable employees to obtain results, thus increasing their self-confidence or effectiveness (Alasiri & Salameh, 2020). Productivity is also improved by the mechanization and digitization of daily jobs. Employees can realize the logic of achievement if they perceive the progressive effects of technology. In other words, it can be said that psychological empowerment inspires workers to feel free and valued in their profession. This ability to liberty promotes enormous problem-solving ideas and creativity, which are essential to give the whole possibility of digital instruments (Afram et al., 2022). When free workers participate in the progressive application of DT and cost value, these consequences positively affect the financial performance of an organization. Psychological empowerment fills the gap between DT adaptability and economic success by improving workers’ inspiration (Afram, 2023).

Some elements affect psychological empowerment in the working environment. This may include leadership provision, training and progress, job strategy, and organizational culture (Malik et al., 2021).Leaders who believe they can create an atmosphere where workers feel free and supported to show problem-solving ideas. Jobs that provide variety and independence and provide new opportunities for employment for good work inspire workers to feel empowered. A culture that increases collaboration promotes a sense of empowerment by permitting workers to make decisions. The outcomes of EP are essential at the individual and organizational levels. Free workers are more confident, show innovative concepts, and have a higher motivation for DT (Liu et al., 2023). Moreover, when workers are proficient, they can demonstrate new problem-solving ideas. PE promotes a high level of job satisfaction, which decreases profit rates.

**Hypothesis 4.**  *PE mediates the link between DT and OFP*.

#### 2.6.5. Innovative Work Behavior and Psychological Empowerment

Employees with IWB exhibit improved competence, can provide creative problem solutions, and can help develop new goods or services. IWBs improve an organization’s effectiveness and offer new possibilities for value creation. Employees stimulated to think out of the box or research with inspiration often catalyze price savings and process development (Mohsin et al., 2021).The correlation between IWB and PE has been a topic of interest in the organizational literature, highlighting the significance of empowering work environments in promoting employee creativity and innovation. Studies indicate that PE, defined as experiencing a sense of competence, autonomy, and meaningfulness in the workplace, is vital for promoting innovative behaviors among employees. Employees who perceive themselves as psychologically empowered are more inclined to participate in IWBs, such as creating novel ideas, problem-solving, and taking initiatives. PE and employee creativity are directly correlated (Ruhl, 1995). These scholars proposed that empowered individuals are more adept at adjusting to change, exploring novel opportunities, and contributing to corporate innovation. These findings emphasize the need to establish working conditions that enable people to fully utilize their creative abilities and stimulate innovation.

Moreover, studies have suggested that the connection between innovative work performance and PE influences factors such as job characteristics, leadership behavior, and organizational culture (Kamselem et al., 2020). Specifically, the connection between PE and employee creativity is strengthened when people view their employment as both challenging and essential. This emphasizes the importance of job design in improving empowerment results. In summary, this study emphasizes the significance of PE in fostering innovative work performance and proposes strategies for organizational interventions that enhance employee empowerment to promote innovation. Supportive leadership, job independence, resources for creativity, and the organizational environment are antecedents of innovative work behavior.

Moreover, when workers have the liberty to make consequential decisions and opportunities to participate in challenging responsibilities, they are more able to elaborate innovative behaviors (Shafique et al., 2020).Workers experienced higher job liberty, development, and a sense of success. PE has positive consequences by fostering employees’ liberty toward innovation in tasks expressively to achieve the goals of the organization, as it brings workers involved in organizational ambitions (Ribeiro et al., 2023).

Thus, the following hypothesis is established.

**Hypothesis 5.**  *IWB has a significant relationship with PE*.

#### 2.6.6. Innovative Work Behavior and Organizational Financial Performance OFP

Work behaviors, including knowledge creation, application, acceptance, and providing support, can increase OFP. A few studies explain this positive effect through improved market share, profits, and expenditure decline. However, recognizing such beneficial behaviors depends on having sympathetic values in the organization, strong management, supply provision, and the capacity to transform these employee efforts into financial outcomes (Choi et al., 2020). Similarly, by elevating the value of innovation, organizations can catalyze their power to achieve economic (Liu et al., 2020).

Therefore, Innovative work performance also relates to OFP. It provides a foundation for organizational development and yields competitive benefits. By adopting the philosophy of vision and flexibility, businesses can connect innovative ideas to update processes, improve efficiency, and continue to succeed in a dynamic marketplace (Carvalho et al., 2019). Workers who are involved in innovative behaviors classify more innovative and effective methods to operate, decrease costs, and improve consumer values. These product improvements may bring about huge profits and strengthen the marketplace position of an organization. Moreover, promoting innovative behavior, improving brand standing, and increasing market assets are all good financial outcomes. Thus, the link between IWB and OFP is reciprocal: as companies enlarge innovative work behavior, they can enhance financial progress and make more capital for innovation (Asbari et al., 2023).

The elements of IWB that involve its positive link with organizational financial performance are organizational elements that promote creativity (Hong & Zainal, 2022). Leadership support is essential for a leader who inspires imagination and gives capital to create an atmosphere where workers feel free to be involved in innovative behaviors. Job independence is another antecedent, as workers are liberal enough to show new ideas with new creative solutions and are more likely to be involved in IWB, thus improving organizational processes (Knezović & Drkić, 2021). IWB can encourage higher costs by optimizing processes and enhancing development. It can also facilitate asset development through new innovative ideas and the exploration of market assets. Tesla Company is an example that promotes IWB by inspiring creative thinking in the renewable energy sector (Mustafa et al., 2023).

Accordingly, the following hypothesis is proposed:

**Hypothesis 6.**  *IWB has a positive relationship with OFP*.

#### 2.6.7. Psychological Empowerment as a Mediator Between Innovative Work Behavior and Organizational Financial Performance

PE is a critical mediator between IWB and OFP. IWB involves creating and executing the inspired concept of employees; IWB increases competitive advantage and influences the organization’s capability to adjust. The link between innovative work behavior and organizational financial performance is supported when it includes psychological empowerment (Almulhim, 2020a). Experienced workers are inspired to use innovative problem-solving ideas, drive limitations in their roles, and increase customer liberty. These innovations also enhance financial performance by restructuring actions, improving facilities, and distinguishing the company in the marketplace. Moreover, when organizations promote psychological empowerment, they create a workplace that inspires workers to fulfill their work and is associated with organizational aims (Al Otaibi et al., 2023).

Psychological empowerment is an essential link between innovative work behavior and organizational financial performance. It inspires workers to use their abilities, positively affecting an organization’s bottom line. Organizations note higher employee creativity and maintenance, and create a base for innovation that brings financial performance by encouraging a culture of empowerment (Nasir et al., 2022). Moreover, organizations that encourage psychological empowerment can imagine important progressive ways in both employee creativity and organizational productivity (Khan et al., 2020).

As a mediator, PE is essential for linking IWB to financial performance. Permitted employees produce inspired ideas, and the organization should consider their application via well-organized procedures and developed goods and services. These results give rise to competitive improvement in the organization or profit development. PE works as an intermediary, determining how financial achievement is achieved by the IWB. Companies create an authorizing atmosphere to see the positive effects of IWB, as authorized employees are located to obtain new materialistic ideas and successfully implement them (Schermuly & Meyer, 2020).

**Hypothesis 7.**  *PE mediates the link between IWB and OFP*.

#### 2.6.8. Smart Technologies as a Moderator Between Psychological Empowerment and Organizational Financial Performance

ST raises an organization’s internal and external relationships and plays a vital role in the organizational construction process. Employees perceive the logic of the capability and importance of their tasks through PE; if individuals can discover more tactics and observe services, they are inspired to develop new organizational advantages. Authorization becomes a motivating force after continued innovation within an organization. STs are the greatest indicators for establishing the best service. This transformation is essential for improving a firm’s performance, including profits. Technology broadly improves a firm’s performance (Edeh et al., 2020). ST works as an essential moderator by highlighting the technology interface gap. PE and independence are technologies like artificial intelligence and data analytics that enhance this empowerment by providing instruments that strengthen progress.

Moreover, the use of innovative technologies provides instruments that enhance productivity. However, ST increases the effect of trained workers on organizational financial performance. Trained workers like to provide more solution-making ideas, while updated ways provide insights that lead to these struggles (George & George, 2023). This decreases operational expenditures, improves consumer satisfaction by adapting facilities, and brings profits (Lim et al., 2020b).

Organizations support the liberation of individuals with vast financial aims by promoting a working atmosphere where workers feel with updated instruments. This symphonic relationship confirms development and places ST as an essential mediator in the social component of a technology-bought period (Kwan et al., 2024).

**Hypothesis 8.**  *ST moderates the link between PE and OFP*.

**Figure 1:**
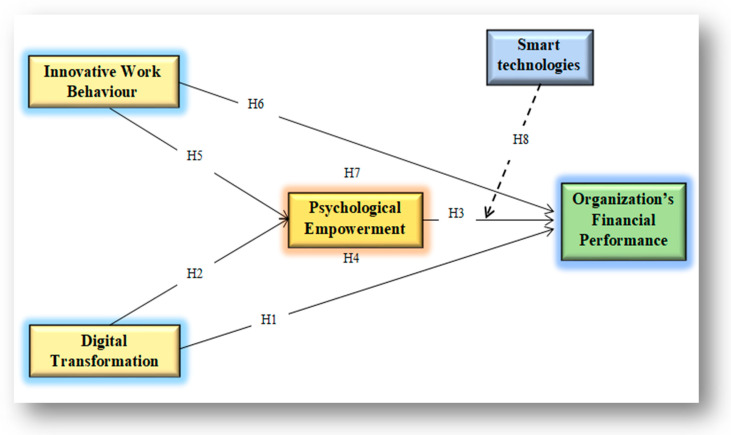
Conceptual framework.

Figure 1 shows the conceptual framework. OFP is the dependent variable, and IWB and DT are the independent variables. Additionally, PE works as a mediator between IWB or OFP, DT, and firm performance (Liu et al., 2019). Finally, ST acts as a moderator that generates a strong link between PE and OFP.

## 3. Methods and Data Collection

### Population and Sample Size

This study was based on a cross-sectional analysis and adopted a convenient data collection sampling technique. Convenience sampling aims to obtain accurate findings as it is a widely used sampling method in social science research (Kianto et al., 2018). Convenience sampling was selected due to practical constraints, such as time and access limitations, which aligned with the objectives of this study. Given the focus on employees directly involved in digital transformation and innovative work behavior, the method allowed efficient access to individuals who could provide valuable insights into the research variables. Thus, the individual holding a position in the top, middle, or lower management was the respondent of this study. The banking sector of Saudi Arabia comprised the population of this study, consisting of 11 local banks (Haque, 2020). The top three banks by market share (Saudi National Bank (SNB), Al Rajhi Bank, and Riyad Bank) were considered for this study. In Saudi Arabia, the banking sector represents three-quarters of the national GDP, with conservative leverage and a substantial capital buffer. This trend is expected to strengthen further with the ongoing expansion of financial services. Furthermore, this sector contributes significantly to national income (Alzahrani & Almazari, 2020).

Furthermore, ethical approval was obtained from the Research Ethics Committee (REC) of the University of Ha’il, Saudi Arabia, before the primary data analysis phase began. Second, in-depth interviews were conducted with five accomplished academic scholars from three universities with research expertise to ensure the accuracy of the questionnaires. Third, 45 participants from five firms were involved in the preliminary testing phase. The screening procedure encompassed multiple stages to detect and resolve the concerns related to the survey.

The research team comprehensively evaluated the questionnaire items to determine their clarity, relevance, and suitability for this study. All unclear or unnecessary items were carefully reviewed and addressed to enhance the overall quality of the questionnaire. Similarly, items that did not meaningfully contribute to the objectives of the questionnaire were eliminated to streamline the survey. It was modified to improve coherence and make it easier for respondents to understand and provide accurate answers. These changes aimed to improve the overall reliability and effectiveness of the questionnaire, ensuring that only relevant and clear items remained. Cronbach’s alpha coefficients were computed for each scale to assess the internal consistency of the items within each construct. To improve the general reliability of the measurements, scales with low internal consistency were either altered or eliminated from the questionnaire.

Moreover, to increase the response rate, the questionnaire was constructed in English (see Appendix A) and Arabic. Back-translation methods were used to ensure the accuracy of the questionnaire. To validate the questionnaire, 500 questionnaires were distributed by hand and via e-mail between 2 August 2023 and 31 December 2023. Respondents ensured that the survey results would only be used for academic purposes and that their responses would be kept confidential. A multi-faceted approach was adopted to ensure that the questionnaires were distributed effectively and to maintain high response rates. First, the target audience was carefully identified, and the questionnaires were distributed through the most appropriate channels, such as e-mail and by hand. A clear and concise introduction was included to explain the purpose of the survey and its importance.

Additionally, reminders were sent to the participants to encourage timely completion. Incentives such as gift cards or the promise of sharing key findings are offered to boost participation. Finally, the questionnaire was designed to be brief and user-friendly, minimizing the time required to complete it, thus improving the likelihood of higher response rates. The survey response rate was 78%, as 391 filled-out questionnaires were received, of which 387 were analyzed. According to Aibinu & Al-Lawati (2010) a response rate of 64% or more is favorable for further processing. Table 1 presents the respondents’ demographic features (Naiwen et al., 2021). All respondents were male due to the lack of representation of females in top management. There were 29.5% of participants with an education less than a bachelor’s degree, 30.8% with a bachelor’s degree, and 39.7% with a degree equal to a master’s or higher. Regarding the participants’ job positions, 25.9% are from top management, 36.5% are related to middle management, and 37.6% are from lower management.

## 4. Results

### 4.1. Measurement Model

The measurement items for the variables are used from previous studies, and all the variables are measured on a Likert scale of 1–5, where 1 indicates strongly agree and 5 means strongly disagree. Further, after the analysis, Table 2 elaborates on Cronbach’s alpha (CA) values. These values met the threshold level given by Hair et al., 2022 of 0.7–0.9. Specifically, OFP has a CA of 0.896, DT has a CA of 0.892, ST has a CA of 0.945, and IWB has a CA of 0.934. The composite reliability (CR) values for each construct are as follows: DT (0.894), IWB (0.948), ST (0.953), PE (0.946), and OFP (0.923). The average variance extracted (AVE) values exceed the threshold level of 0.5: DT (0.751), IWB (0.583), ST (0.706), PE (0.693), and OFP (0.951).

### 4.2. Fornell−Larcker Criterion

For discriminant validity related to constructs, the Fornell−Larcker criterion, which is the square root of AVE, is used. Specifically, this value should be greater than the AVE of the other constructs (Ab Hamid et al., 2017; Hair & Alamer, 2022) Table 3 presents the results of the Fornell–Larcker criterion.

In the current model, 0.02 indicates a minor effect, 0.15 signifies a moderate effect, and 0.35 represents a substantial effect. The significance of the values indicates that PE exhibits a minor effect, with a value of 0.034, whereas OFP demonstrates a moderate effect, with a value of 0.214. Additionally, Q^2^ within the model elucidates predictive relevance as a measure of out-of-sample predictive power (Abbasi et al., 2024). The Q^2^ values are 0.154 for PE and 0.376 for OFP, and both are greater than zero, indicating predictive relevance, as shown in Table 4. The Q^2^ value of 0.154 for PE shows a small positive predictive accuracy, while the higher Q^2^ value of 0.376 for OFP demonstrates a more substantial predictive power for organizational performance. According to general benchmarks, a Q^2^ value greater than 0 indicates the model’s ability to predict, with values closer to 0.35 or higher often regarded as strong for predictive relevance. Therefore, the Q^2^ values confirm that the model has valid out-of-sample predictive capacity. In PLS-SEM (Partial Least Squares Structural Equation Modeling), the Variance Inflation Factor (VIF) is crucial for detecting multi-collinearity among predictor variables. Monitoring VIF values helps ensure model stability and interpretability by highlighting when predictors are too similar, which could lead to unreliable coefficient estimates. Acceptable VIF values are typically below five, indicating low to moderate multi-collinearity. Values between 5 and 10 suggest moderate multi-collinearity, which may require attention but is not always problematic (Kasraei et al., 2024). However, VIF values above 10 indicate high multi-collinearity, which could compromise the model’s validity. In such cases, researchers may need to remove or combine redundant predictors. In the current study, the VIF value is 1; therefore, researchers can enhance their PLS-SEM models’ predictive accuracy and robustness.

Furthermore, the R^2^ values of 0.760 for psychological empowerment (PE) and 0.735 for organizational financial performance (OFP) indicate the proportion of variance explained by the model for these constructs. In other words, these values reflect how well the independent variables in the model describe the variability in PE and OFP. According to Falk and Miller’s criterion, an R^2^ value of 0.10 or higher can explain a meaningful amount of variance. The R^2^ value of 0.760 for PE suggests that the model explains 76% of the variance in psychological empowerment, which represents strong explanatory power. This means that the predictors used in the model (such as digital transformation and innovative work behavior) significantly influence PE.

Similarly, the R^2^ value of 0.735 for OFP indicates that the model explains 73.5% of the variance in organizational financial performance. This also demonstrates strong explanatory power, indicating that the variables in the model account for a substantial portion of an organization’s financial outcomes. In short, these high R^2^ values suggest that the model provides a strong explanation for both Psychological Empowerment and Organizational Financial Performance, indicating that the independent variables included effectively explain the variability in these constructs. We also evaluate the standardized root mean square residual (SRMR) to assess model fitness. The SRMR value is 0.059, below the threshold of 0.08, indicating a good fit. A lower SRMR value suggests a more minor discrepancy between the predicted and observed data, indicating a better fit. A value below the commonly accepted threshold of 0.08 signifies that the model fits the data well, meaning that the residuals (or differences) between the observed and predicted values are minimal. When the SRMR is below this threshold, it suggests that the model’s structure adequately represents the data, supporting its validity and reliability.

### 4.3. Assessment of Structural Model

**Figure 2:**
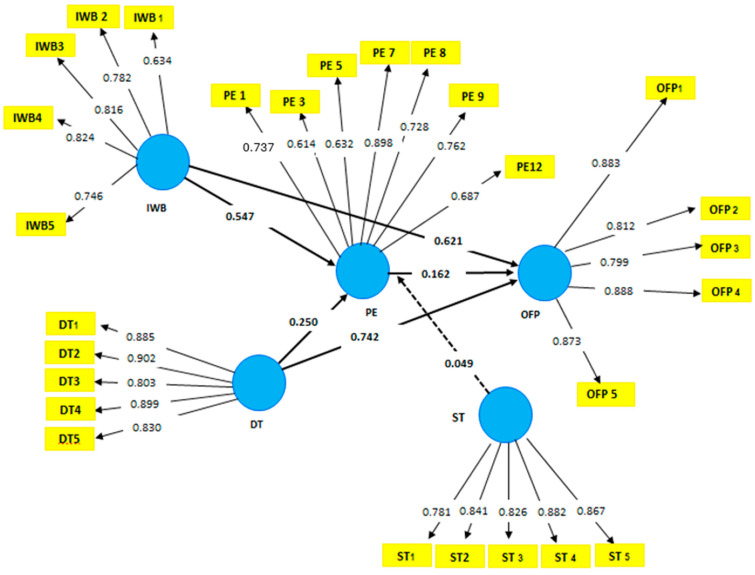
Path analysis.

**Figure 3:**
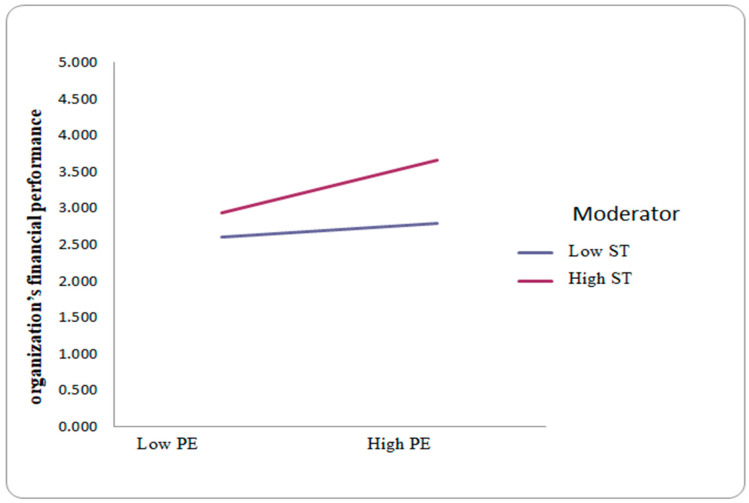
Smart technology (ST) moderates the relationship between workers’ psychological empowerment (PE) and organizational financial performance (OFP).

The structural model findings (Table 5, Figure 1) indicate that DT has a positive and significant relationship with OFP. Additionally, PE demonstrates a positive relationship with OFP, β=0.16. Further, PE positively mediates the relationship between DT and OFP, with β = 0.14. Moreover, PE serves as a significant mediator between IWB and OFP. Finally, ST moderates PE and OFP, significantly enhancing the relationship between IWB and OFP, as shown in Figure 2. Further, based on the “*p*” value, this study accepts all hypotheses. Table 5 presents the values for each variable in detail and moderating effect is presented in Figure 3.

### 4.4. Discussion

This research framework is designed to follow an innovative business model and social cognitive theories (Aure et al., 2019). Data analysis reveals that DT has a positive and consequential link with OFP in Saudi Arabia’s financial sector. Digital transformation is not only necessary for revenues, but also, in the current century, an ingredient of survival. No sector without digital adaptation can survive or compete in the market. Studies (Ikeda & Marshall, 2016; Salampasis & Mention, 2018) have emphasized the beneficial effects of IWB on organizational performance, stressing the role of employee creativity and proactive behavior in fostering innovation results. Our results align with previous research and show a strong positive correlation between IWB and OFP, highlighting the need to encourage an innovative culture within businesses.

Further, integrating digital technologies into all industry characteristics significantly influences OF (Hussain et al., 2022)—DT results in increased profit and market segmentation by improving consumer engagement and operational productivity (Azam et al., 2023). Moreover, organizations leverage DT as a strong instrument for financial development and competitive benefits by gaining the advantages of financial systems, empirical executives, and upgraded consumer correlations. Digital transformation drives increased revenue, market share, and reduced costs by leveraging advanced technologies to streamline operations, enhance customer experience, and optimize decision-making. Further, businesses can improve efficiency and scalability by integrating tools like artificial intelligence (AI), cloud computing, big data analytics, and the Internet of Things (IoT) while minimizing operational inefficiencies. AI and data analytics provide insights into customer behavior, allowing personalized marketing strategies that boost sales and expand market reach. Cloud computing reduces infrastructure costs by offering scalable solutions, while automation technologies reduce labor expenses and increase productivity. Moreover, digital transformation enables businesses to rapidly adapt to market changes, innovate faster, and tap into new revenue streams, all of which contribute to competitive advantage and long-term profitability. These results are in line with previous studies.

Recent research indicates that DT crucially impacts the PE of employees by redesigning the work atmosphere (Mohsin et al., 2024). The transformative implications of technology adoption on organizational processes and outcomes have also been highlighted in the literature on DT (Popovič et al., 2018). Further, digital tools enable more remote work, flexible hours, and decentralized decision-making, and employees gain greater control over their tasks and work environment. This sense of autonomy is measured through employee surveys that assess perceived decision-making authority, control over work methods, and the ability to self-manage. In this study, these factors are linked to greater job satisfaction and motivation, as digital platforms facilitate seamless collaboration, real-time communication, and personalized workflows. Specific aspects of the work environment most affected by digital transformation include communication channels, project management tools, and the structure of team dynamics, all of which contribute to a more empowered and flexible workforce capable of driving innovation and achieving better performance outcomes (Hussain et al., 2022).

Furthermore, ST has a positive moderating effect on PE. Access to real-time information and data analytics enhances financial sector employees’ abilities to make informed decisions, thus fostering a sense of competence. The linking of employees in the decision-making procedure shows the importance of DT as an effective organization, supporting the logic of possession and effect over continuing dynamics. Generally, DT can affect employees’ PE by generating more change, elasticity, and a cooperative work atmosphere. Moreover, constant skilling opportunities help employees in their individual and professional development (Yasin et al., 2022). The mechanization of routine responsibilities brings productivity gains, permitting employees to divert their attention to more tactical and significant work. In addition, incorporating the ST into the financial sector improves income productivity.

From a theoretical perspective, this study has used theories such as business model innovation and social learning theory. The positive relation of variables shows that business model innovation and social learning theory provide a strong framework to justify the positive relationship between digital transformation and financial performance. Business model innovation involves rethinking and redesigning delivered and captured digital technologies to streamline processes, reduce costs, and create new revenue opportunities (Ranta et al., 2021; Yasin et al., 2022). By adopting digital tools like automation, artificial intelligence, and data analytics, businesses innovate their models to be more agile and customer-centric, leading to improved financial outcomes. Social learning theory complements this by highlighting how individuals and organizations learn by observing others. As companies embrace digital transformation, they also engage in a cycle of learning and adaptation, improving their ability to innovate and compete. Through social learning mechanisms, organizations replicate successful digital strategies from industry leaders, enhancing their operational efficiency, market reach, and financial performance. Combining business model innovation and continuous learning through digital practices leads to sustainable growth and financial success (Ahsan, 2024).

#### 4.4.1. Practical Contributions

The research findings indicate that the selected set of variables plays a fundamental role in the banking system, whether the nature of the impact is positive or negative. The employer’s motive DT, ST, and OFP can be achieved easily with the encouragement of employees and maintaining the induction of employees in IWBs. The employer’s financial goals are based on capturing maximum capital from the market and achieving high profitability, which comes from the implementation of ST. The first-time installation of a system is not a significant task, but maintenance. The business’s competitive advantages can be achieved by maintaining ST, DT, and OFP. This research suggests that low-cost online and mobile banking facilities can provide a boost to the banking sector’s performance because of the accessibility of consumers anywhere and anytime. Banking sector employees should be trained to provide opportunities for innovative work. Innovative facilities should be provided to employees within affordable ranges to boost their morale and work for publicity purposes.

Furthermore, the findings emphasize the necessity of encouraging IWB and adopting DT to improve corporate financial performance (CFP) in the digital age. To remain competitive and sustainable in today’s rapidly changing business world, organizations should emphasize efforts to foster an innovative culture and invest in digital technologies. By acknowledging PE’s mediating function, practitioners can focus on empowering people to drive innovation and effectively use digital tools to achieve strategic goals. Policymakers are encouraged to support efforts that foster industry-wide innovation and digitization, such as stimulating R&D investments. Furthermore, governments should prioritize policies that promote workforce development and digital literacy to ensure that companies and individuals are prepared to face the challenges and opportunities of the digital economy. Service organizations like the banking sector need to pay attention to enhancing employees’ psychological empowerment (PE), which is considered an essential prerequisite for IWB. PE is a moderating variable that offers practical solutions for the improvement of IWBs at the sectorial and individual levels. The promoting work of IWBs of employees can stimulate managers of banks to improve PE. A complete understanding of sectorial leaders regarding the antecedents of IWBs, implementation of DT and ST as well as protection of employees’ PE to stimulate employees’ IWBs. In the running century, leaders should be fully aware of employees’ intrinsic motivation and compatibilities and empower them to decide the implementation of ST and OFP.

#### 4.4.2. Limitations and Future Research Suggestions

This study has different limitations. These limitations permit deliberation. Firstly, the analysis is based on a particular period and regulates the possibility of study. The classification of progressive policies, which are critical for effective digital supply chain management and the capability to investigate the subject matter, is hindered by a lack of longitudinal data analysis. Secondly, the data were collected from a single country, Saudi Arabia, and the data analysis was conducted on a general basis. A more comprehensive method is suggested to address these limitations in future research. This study can select employees from different departments to provide more enhanced and varied perspectives to this model. A more diverse geographical sample could provide a more comprehensive understanding because it captures the variations in cultural, economic, and social contexts that influence behavior, attitudes, and outcomes. Different regions may experience unique challenges or benefits related to the study topic, which offers a broader perspective on the factors that drive specific trends. Including participants from multiple geographical areas makes the research more generalizable and can reveal patterns in a more homogeneous sample. This diversity also helps identify region-specific solutions or strategies, enriching the study’s findings and practical applications.

Thirdly, this study is cross-sectional. The study suggests that future research should use longitudinal data for the same model, which can significantly affect the robustness and generalizability of the findings. This data type would be beneficial for studying digital transformation’s impact on psychological empowerment, innovative work behavior, and financial performance. Without longitudinal data, capturing changes and trends over time is challenging and may result in overlooking the long-term effects of digital transformation. For example, the immediate positive impact on financial performance may not reflect potential adaptation challenges or diminishing returns that could emerge later.

Similarly, short-term boosts in employee autonomy and flexibility may not account for how these factors evolve as employees become more accustomed to new technologies or as organizational structures change in response to digital transformation. Without tracking these dynamics over time, the findings may offer only a snapshot rather than a comprehensive understanding of how digital transformation influences empowerment and performance. Additionally, the absence of longitudinal data limits the ability to generalize the results across different timeframes and contexts, as it is unclear whether the observed outcomes are sustainable or context-specific. Furthermore, the respondents of this study are only male due to the limited number of female employees in the banks in Saudi Arabia; as mentioned by Alqahtani (2023) the representation of women in top management in Saudi Arabia is only 1.3%. Therefore, future studies can use the same framework to collect data from male and female participants.

Lastly, the study’s endogenous variable is the bank’s performance, measured in the market segment and development. However, future research could consider other metrics, such as commercial trademarks, image, average productivity, or other endogenous variables, to better understand firm progress. This would contribute to a better and more varied assessment of the factors persuading financial performance and their impact on overall business outcomes.

## 5. Conclusions

In this technological and innovation era, the transformation of the financial sector generated high-level revenue and facilitated the business’ customers. This research has explored the relationship between innovative behavior (IWB), digital transformation (DT), and organizational financial performance (OFP). At the same time, worker’s psychology empowerment (PE) and smart technology (ST) are considered mediating variables. The variables were selected to gain a deep insight into sectorial growth and the leading interest of both parties (Employer and Employees) using the structural equation modeling, least squares (SEM-PLS) approach. The result of this research work is the prediction that IWB positively relates to OFP. The logical standing of this relationship is also justifiable because innovative work behavior (IWB) for sure enhances organizational financial performance (OFP). Innovative work can captivate the customer and capture the maximum possible market compared to old-fangled and old-tradition-based work. The positive relationship between IWB and OFP is moderated by employees’ psychology empowerment (PE). Employees’ psychological empowerment can increase the chances of accepting innovative work behavior, which leads to upward financial performance of the organization and revenues. Digital transformation positively influences organizational financial performance (OFP) by mediating smart technology (ST). In terms of practical significance, while PE has a minor effect on organizational outcomes, it still plays a crucial role in shaping employee motivation and engagement. A minor effect does not imply insignificance; even a small increase in PE can improve employee well-being, job satisfaction, and commitment. This translates into a more positive work environment, better team dynamics, and potentially lower turnover rates. From a practical standpoint, organizations should not overlook the value of fostering PE through supportive leadership, training programs, and employee development initiatives.

On the other hand, the moderating effect of organizational performance highlights its more direct and impactful relationship with key outcomes such as profitability, productivity, and long-term sustainability. A moderate effect indicates that improvements in organizational processes, resources, and performance management strategies can significantly enhance a company’s competitive advantage. Therefore, businesses should prioritize optimizing operational efficiency, setting clear performance metrics, and promoting a culture of continuous improvement to realize tangible benefits. While PE provides foundational support for employee-level improvements, organizational performance drives broader business success. Although both are crucial, their contributions differ in terms of scale and scope. Therefore, this research can be beneficial for the banking sector of Saudi Arabia, and practical implications help to increase financial performance by carrying the best positive image of the banking sector, keeping employees, and providing maximum satisfactory services to banks’ customers.

## Figures and Tables

**Table 1:** Participant profile.

Variable Name	Classification	Percentage
Age	Less than 30	30.6
	30–45	29.4
	45–55	23.8
	Greater than 50	16.2
Degree	>bachelor	29.5
	Bachelor	30.8
	Master or above	39.7
Gender	Male	100
	Female	0
Management position	Top	24.9
	Middle	38.5
	Lower	36.6
Experience	>1	21.4
	1 to 5	25.2
	6 to 10	24.6
	11–20	15.5
	<than 20	13.3

**Table 2:** Measurement model.

Latent Variable	Items	Factor Loadings	CA	AVE	CR
Digital transformation	DT 1	0.885	0.892	0.751	0.894
	DT 2	0.902			
	DT 3	0.803			
	DT 4	0.899			
	DT 5	0.830			
Innovative work behavior	IWB 1	0.634	0.934	0.583	0.948
	IWB 2	0.782			
	IWB 3	0.816			
	IWB 4	0.824			
	IWB 5	0.746			
Smart technologies	ST 1	0.781	0.945	0.706	0.953
	ST 2	0.841			
	ST 3	0.826			
	ST 4	0.882			
	ST 5	0.867			
Psychological empowerment	PE 1	0.737	0.935	0.693	0.946
	PE 3	0.614			
	PE 5	0.632			
	PE 7	0.898			
	PE 8	0.728			
	PE 9	0.762			
	PE 12	0.687			
Organizational financial performance	OFP 1	0.883	0.896	0.951	0.923
	OFP 2	0.812			
	OFP 3	0.799			
	OFP 4	0.888			
	OFP 5	0.873			

**Table 3:** Fornell−Larcker criterion results.

	DT	IWB	PE	ST	OFP
DT	0.869				
IWB	0.868	0.867			
PE	0.852	0.852	0.833		
ST	0.738	0.768	0.714	0.830	
OFP	0.849	0.859	0.812	0.788	0.841

**Table 4:** Results of saturated.

Variable	R^2^	Adjusted R Square	VIF	Q^2^	F^2^	SRMR
PE	0.760	0.757	1.289	0.154	0.034	0.059
OFP	0.735	0.734	1.336	0.376	0.214	

Note: Variance inflation factor (VIF); predictive relevance (Q^2^); effect size (F^2^); standardized root mean square (SRMR); determination of coefficient (R^2^).

**Table 5:** Hypothesis testing results.

Construct	Beta	Sample Mean (M)	Standard Deviation	*T* Statistics	*p* Values	Decision
DT → OFP	0.742	0.744	0.053	14.060	0.000	Accepted
DT → PE	0.250	0.256	0.085	2.945	0.003	Accepted
PE → OFP	0.162	0.157	0.069	2.355	0.019	Accepted
DT → PE → OFP	0.144	0.143	0.046	3.127	0.002	Accepted
IWB → PE	0.547	0.545	0.075	7.330	0.000	Accepted
IWB → OFP	0.621	0.325	0.065	7.310	0.009	Accepted
IWB → PE → OFP	0.144	0.143	0.046	3.127	0.002	Accepted
ST × PE → OFP	0.049	0.048	0.025	1.943	0.052	Accepted

## Data Availability

The authors confirm that the data supporting the findings of this study are available as Appendix A.

## Appendix A

Measurement of variables

Digital Transformation

DT has the following items:

- Within the organization, our objective is to enhance connectivity among various business processes by integrating digital technologies.
- Within the organization, our goal is to facilitate information exchange through digital means.
- Within the organization, our focus is on digitizing all aspects that can be transformed into digital formats.
- Within the organization, we gather extensive amounts of data from various sources.
- Within the organization, our objective is to improve the efficiency of customer interfaces through digital enhancements and technologies.

Innovative Work Behavior

Five items measure this variable (Almulhim, 2020a; Liu et al., 2016; Pian et al., 2019):

- Normally, I produce inventive ideas and considerations during my work.
- I endorse my novel ideas with coworkers and leaders, looking for their support and gratitude.
- To bring my ideas or innovations to fruition, I have made concerted efforts to secure the necessary resources.
- I proactively develop suitable plans or projects to implement my innovative ideas.
- I consistently offer recommendations to assist my colleagues in bringing innovative ideas to fruition.

Psychological Empowerment

PE acts as a mediator and creates the relationship between DT, IWB and OFP. Twelve items are adopted from Almulhim (2020b) to evaluate this

- Smart Technologies
- Seven items measure ST:
- In the organization, every device is designed to be programmable.
- In the organization, all devices can both send and receive messages.
- All the devices within the organization can record and store all information.
- Within the organization, each device can be uniquely identified.
- Within the organization, all devices can respond to changes in their environment.
- Within the organization, all devices can record and store all information.
- Within the organization, all devices can identify and interact with other entities.

Organizational Financial Performance

The following items are used for the financial performance of the organization:

- ROA
- ROE
- Return on sales.
- Organization’s market share
- Sales growth
